# 伊立替康二线治疗难治性复发小细胞肺癌的疗效分析

**DOI:** 10.3779/j.issn.1009-3419.2021.103.04

**Published:** 2021-03-20

**Authors:** 贺 邢, 洁 张, 凤娟 葛, 昕航 余, 会敏 边, 福亮 张, 健 方

**Affiliations:** 1 36100 吉林，吉林国文医院肿瘤内科 Department of Oncology, Jilin Guowen Hospital, Jilin 136100, China; 2 100142 北京，北京大学肿瘤医院暨北京市肿瘤防治研究所胸部肿瘤内二科，恶性肿瘤发病机制及转化研究教育部重点实验室 Department Ⅱ of Thoracic Oncology, Key Laboratory of Malignant Tumor Pathogenesis and Translational Research (Ministry of Education), Peking University Cancer Hospital & Institute, Beijing 100142, China

**Keywords:** 肺肿瘤, 难治性复发, 伊立替康, 耐药, 疗效分析, Lung neoplasms, Refractory relapse, Irinotecan, Drug resistance, Analysis of curative effect

## Abstract

**背景与目的:**

肺癌死亡率在恶性肿瘤中高居首位，小细胞肺癌（small cell lung cancer, SCLC）是肺癌恶性程度最高的一种，其倍增时间短，患者在治疗过程中易发生迅速耐药，且复发后病情迅速恶化。目前除拓扑替康外，缺乏有效的二线单药化疗方案。本研究旨在探究伊立替康（irinotecan, CPT-11）二线单药治疗难治性复发SCLC的疗效及安全性。

**方法:**

收集吉林国文医院肿瘤内科2012年4月-2020年3月确诊的一线治疗后6个月内复发的经单药CPT-11二线化疗的SCLC患者107例，末次随访至2020年11月。记录患者的无进展生存时间（progression free survival, PFS）和总生存时间（overall survival, OS），观察单药CPT-11化疗效果以及不良反应发生情况。

**结果:**

患者的中位PFS为3.8（3.4-4.4）个月，中位OS为8.1（6.5-10.9）个月，客观缓解率（objective response rate, ORR）为16.82%（18/107），DCR为55.14%（59/107），3级-4级不良反应发生率较低，其中中性粒细胞减少为13.08%，迟发性腹泻为7.48%，恶心呕吐为17.76%，肝功能受损为6.54%。影响单药CPT-11二线化疗患者PFS的因素有性别（*P*=0.001）、NSE（*P*=0.029）以及积液状态（*P*=0.040），影响OS的因素仅为NSE水平（*P*=0.033）。

**结论:**

针对于难治性复发的SCLC患者，CPT-11单药二线化疗方案有一定疗效，耐受较好，值得推广应用。

## Abstract (en)

**Background and objective:**

Among malignant tumors, lung cancer has the highest mortality rate. Small cell lung cancer (SCLC) is a kind of malignant lung cancer. Its doubling time is very fast. Patients are prone to drug resistance during treatment, and their condition often deteriorates rapidly after recurrence. Except for topotecan, there is a lack of effective second-line single-agent chemotherapy. This study aims to analysis the efficacy and safety of irinotecan (CPT-11) in the second-line treatment of refractory and relapsed SCLC.

**Methods:**

A total of 107 SCLC patients were collected from the Department of Oncology, Jilin Guowen Hospital, who were diagnosed from April 2012 to March 2020, relapsed within 6 months after first-line treatment, and received second-line chemotherapy with single-agent CPT-11. Follow-up until November 2020, calculate the patient's progression free survival (PFS) and overall survival (OS), and summarize the effects and adverse reactions of CPT-11 chemotherapy.

**Results:**

The patient's median PFS was 3.8 (3.4-4.4) months, median OS was 8.1 (6.5-10.9) months, objective response rate (ORR) was 16.82% (18/107), and DCR was 55.14% (59/107). The incidence of grade 3-4 adverse reactions in patients was relatively low. Among them, neutropenia was 13.08%, delayed diarrhea was 7.48%, nausea and vomiting was 17.76%, and liver function impairment was 6.54%. The influencing factors of PFS in single-agent CPT-11 second-line chemotherapy were gender (*P*=0.001), NSE (*P*=0.029), and effusion (*P*=0.040). While the influencing factors of OS were NSE level only (*P*=0.033).

**Conclusion:**

For patients with refractory relapsed SCLC, CPT-11 single-agent second-line chemotherapy has a certain effect, is well tolerated, and is worthy of promotion.

肺癌是临床中最常见的恶性肿瘤之一，其死亡率在全球范围内恶性肿瘤中高居首位。根据病理特征可将其分为小细胞癌（small cell lung cancer, SCLC）和非小细胞癌（non-small cell lung cancer, NSCLC），其中SCLC具有倍增时间快，易发生迅速耐药性的特点，患者更加难以治疗^[1-3]^。研究资料^[4-8]^显示，处于广泛期SCLC患者疾病复发率超过90%，且复发后患者病情常常迅速恶化。目前除拓扑替康外，仍缺乏有效二线治疗方案。为探讨伊立替康（irinotecan, CPT-11）的临床治疗效果，本研究选取我院真实世界中收治的107例难治性复发SCLC，采取CPT-11进行二线化疗，观察CPT-11单药化疗的疗效。

## 1 对象与方法

### 1.1 病例选择

收集吉林国文医院肿瘤内科自2012年4月-2020年3月期间确诊的一线治疗6个月内复发（转移）SCLC患者。纳入标准：①组织学或细胞学确诊为小细胞肺癌，且一线治疗后6个月复发（转移）患者；②患者的Karnofsky功能状态评分高于60分；③至少完成2个周期化疗，预计生存 > 3个月；④对CPT-11无过敏且无明显化疗禁忌证；⑤治疗依从性高，能够配合接受定期随访；⑥应用CPT-11单药进行二线治疗，具有至少一个可评价病灶；⑦患者二线化疗一段时间后出现明显的二线进展。排除标准：①各种原因引起的肝功能异常，存在肝肾功能严重障碍患者；②各种原因导致治疗前即出现外周血血细胞减少，造血功能不足，患有骨髓功能低下的患者；③合并患有严重心脑血管疾病；④患有精神类疾病，无法配合研究；⑤合并其他恶性肿瘤或免疫系统、血液系统疾病；⑥处于妊娠或哺乳期妇女。共计纳入一线治疗6个月内复发的SCLC患者107例，进行回顾性分析。

### 1.2 治疗方法

所有患者采用依托泊苷+顺铂一线化疗方案，二线化疗均采用CPT-11（海南锦瑞制造有限公司，国药准字H20143126，药品规格100 mg）单药，60 mg/m^2^静脉滴注，按照d1、d8、d15，每28天为1个治疗周期，中位治疗周期时间为3（2-6）个疗程，化疗直至疾病进展或患者出现不可耐受的不良反应。

### 1.3 观察指标和药效评定

末次随访至2020年11月30日，统计患者经单药CPT-11治疗后的无进展生存期（progression free survival, PFS）和总生存期（overall survival, OS）。采用美国东部肿瘤协作组（Eastern Cooperative Oncology Group, ECOG）体能状态评分标准评估患者的体力评分情况：活动自如完全正常视为0级；从事较重的体力活动受限但可以自由走动和从事包括一般家务或办公的轻体力活动视为1级；能自由走动及生活自理，但已丧失工作能力视为2级。按照实体肿瘤的疗效评价标准（Response Evaluation Criteria in Solid Tumors, RECIST）1.1版，对药物治疗效果进行评价，包括完全缓解（complete response, CR）、部分缓解（partial response, PR）、疾病进展（progressive disease, PD）和疾病稳定（stable disease, SD）。计算CPT-11治疗后药物客观缓解率（objective response rate, ORR）以及疾病控制率（disease control rate, DCR）。统计患者治疗期间3级以上不良反应的发生情况，包括迟发性腹泻、肝功能异常、呕吐、中性粒细胞减少等。

### 1.4 统计检验

将所有患者数据输入SPSS 22.0软件进行处理，计量资料用均数±标准差（Mean±SD）表示，计数资料用百分率（%）表示，进行单样本*Binomial*检验、单样本*Kolmogorov-Smirnov*检验以及多元*Cox*生存分析。采用GraphPad v6进行*Log-rank*检验，同时绘制患者的*Kaplan-Meier*生存曲线。*P* < 0.05为差异有统计学意义。

## 2 结果

### 2.1 患者基线资料

对所有入组患者一般资料的进行统计，总计确认107例SCLC患者为研究对象，其中男67例，女40例，患者主要为男性（*P*=0.012），患者年龄23岁-76岁，平均年龄为（56.51 ± 9.72）岁，其中年龄 > 65岁患者占比22.4%，≤65岁占比77.6%，年轻患者居多（*P* < 0.001）。处于局限期患者57例（53.2%），广泛期患者50例（46.7%），分期情况并无明显差异。此外，这些患者好发各个部位的肿瘤转移（*P* < 0.001），大多数患者ECOG评分为1分（*P* < 0.001）。详见表 1。

**1:** 入组患者的一般临床信息 General clinical information of patients

Index		*n*	%	*P*
Gender	Male	67	62.6	0.012
	Female	40	37.4	
Age (yr)	> 65	24	22.4	< 0.001
	≤65	83	77.6	
ECOG PS	0	11	10.3	< 0.001
	1	67	62.6	
	2	29	27.1	
Stage	Limited	57	53.2	0.170
	Extensive	50	46.7	
Intrapulmonary metastasis	No	14	13.1	< 0.001
	Yes	93	86.9	
Brain metastases	No	22	20.6	< 0.001
	Yes	85	79.4	
Bone metastases	No	19	17.8	< 0.001
	Yes	88	82.2	
Liver metastases	No	17	15.9	< 0.001
	Yes	90	84.1	
Effusion	No	75	70.1	< 0.001
	Yes	32	29.9	
EOCG: Eastern Cooperative Oncology Group; PS: performance status.				

### 2.2 伊立替康的治疗效果

CPT-11二线单药化疗后，患者中位PFS为3.8（3.4-4.4）个月，中位OS为8.1（6.5-10.9）个月，见图 1。治疗效果：PR 18例（16.82%），SD 41例（38.32%），PD 48例（44.86%），ORR为16.82%（18/107），DCR为55.14%（59/107）。

**1:**
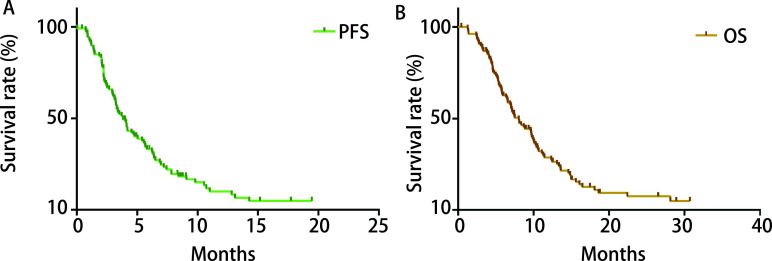
入组患者的生存情况。A：患者无进展生存时间；B：患者总生存时间。 Survival status of the enrolled patients. A: patient's PFS; B: patient's overall survival time. PFS: progression free survival.

### 2.3 不良反应发生率

3级-4级的不良反应发生率较低，其中中性粒细胞减少为13.08%、迟发性腹泻为7.48%、恶心呕吐为17.76%、肝功能受损为6.54%，详见表 2。

**2:** 患者不良反应发生情况 The occurrence of adverse reactions in patients

Adverse reactions	All	Grade 3-4
Neutropenia	22 (20.56%)	14 (13.08%)
Delayed diarrhea	17 (15.89%)	8 (7.48%)
Sick and vomit	36 (33.64%)	19 (17.76%)
Liver and kidney function injury	12 (11.21%)	7 (6.54%)

### 2.4 患者随访生存情况

以患者生存情况包括生存时间和生存状态为因变量，将患者的所有临床指标视为分析的自变量，而指标本身男/女，CEA及NSE数值的大小视为变量的变化值，进行*Cox*多因素回归分析，探究单药CPT-11化疗患者PFS及OS的影响因素，结果显示，患者PFS的影响因素有性别（*P*=0.001）、NSE水平（*P*=0.029）以及积液情况（*P*=0.040），患者OS的因素影响仅有神经元特异性烯醇化酶（neuron-specifific enolase, NSE）水平（*P*=0.033），详见表 3。

**3:** *Cox*生存分析107例SCLC患者生存情况 *Cox* analysis of 107 SCLC survival status

Influencing factors	PFS				OS		
	B	SE	*P*		B	SE	*P*
Gender	1.152	0.355	0.001		0.489	0.330	0.138
Age (> 65 yr)	-0.545	0.555	0.326		0.619	0.528	0.241
ECOG	0.090	0.298	0.762		0.010	0.316	0.975
CEA	-0.004	0.007	0.566		-0.007	0.009	0.450
NSE	0.007	0.003	0.029		0.007	0.003	0.033
LDH	0.000	0.002	0.883		0.000	0.003	0.964
Ki67	0.486	1.355	0.720		1.644	1.338	0.219
Intrapulmonary metastasis	-0.343	0.542	0.526		0.098	0.455	0.829
Supraclavicular LN	0.632	0.454	0.163		0.213	0.436	0.624
Mediastinum LN	0.642	0.614	0.296		0.022	0.572	0.970
Brain metastases	0.175	0.444	0.693		0.758	0.441	0.086
Bone metastases	-0.262	0.517	0.612		0.239	0.532	0.654
Liver metastases	0.703	0.499	0.159		0.119	0.523	0.820
Effusion	0.745	0.363	0.040		0.466	0.391	0.233
First-line local treatment	0.443	0.345	0.199		0.585	0.361	0.105
CEA: carcinoembryonic antigen; NSE: neuron-specific enolase; LDH: lactate dehydrogenase; LN: lymph node.							

## 3 讨论

SCLC恶性程度高，患者一线治疗后易快速耐药，NCCN临床指南定义6个月以内复发的SCLC为难治性小细胞肺癌^[9-15]^。二线单药化疗唯一有证据的为拓扑替康，邢镨元^[16]^研究结果显示，拓扑替康ORR可达7.7%，DCR可达46.2%，患者中位PFS为3个月，3级-4级毒副作用发生也较低。目前其他药物二线单药化疗的证据匮乏，急需加以研究。

韩忠诚^[17]^研究显示，CPT-11和拓扑替康的单药二线化疗的近期及远期疗效相似，提示CPT-11可以潜在作为拓扑替康替代用药。张良^[18]^研究表明，拓扑替康单药化疗中位PFS为3.2个月，中位OS为13.1个月。本研究具体分析CPT-11二线单药化疗难治性复发SCLC患者的疗效及安全性，结果显示，与拓扑替康单药化疗相比，CPT-11具有更好的综合疗效。Shi^[19]^研究显示采用CPT-11治疗不增加患者不良反应，与本研究CPT-11药物耐受较好结果一致。韩森^[20]^研究显示CPT-11是否联合顺铂进行二线化疗的总体疗效相似，ORR为13.0%，DCR为53.7%，中位PFS为2.5个月，与本研究药效结果相似，但比本研究中ORR为16.82%，DCR为55.14%效能偏低，分析原因，本研究纳入的病例数目（107例）2倍多于韩森的研究（54例），队列大小可能是出现结果差异的主要原因，与此同时，一方面可能由于韩森研究中的部分患者联合顺铂进行化疗，虽然CPT-11单药治疗或联合顺铂未在其研究中显现统计学差异，但可能对总体存在细微的影响，另一方面本研究纳入的患者偏向于年轻化（≤65岁占比77.6%），可能因此导致患者耐受较好，治疗效果得以提升。与此同时，本研究进一步分析影响CPT-11单药二线化疗患者的PFS及OS危险因素，结果表明，PFS的影响因素有性别、NSE以及积液有无（*P* < 0.05），OS的影响因素仅有NSE水平（*P* < 0.05）。这些指标在临床工作中，具有潜在风险指示作用。

此外，李旭^[21]^研究结果显示，白蛋白结合型紫杉醇单药化疗对鳞癌的化疗效果较好，但对于SCLC，则显得治疗效果不足。远不及CPT-11单药化疗效果。对于NSCLC的患者，单药吉西他滨与单药紫杉醇均具有较好的临床效果，但缺少单药吉西他滨、单药多西他赛等二线治疗复发型SCLC的应用^[22]^。目前SCLC的相关临床及病理研究均相对较少，需引起足够的重视。

综上所述，CPT-11单药化疗针对于一线治疗6个月内复发的难治性复发SCLC，有一定疗效，患者生存情况较好且毒性较低耐受较好。CPT-11与拓扑替康疗效相似，可作为潜在的拓扑替康替代化疗药物。本研究进一步丰富了CPT-11单药在真实世界的临床研究数据，CPT-11单药作为二线治疗方案值得进一步推广应用。
